# Color Index of Transformer Oil: A Low-Cost Measurement Approach Using Ultraviolet-Blue Laser

**DOI:** 10.3390/s21217292

**Published:** 2021-11-02

**Authors:** Muhamad Haziq Hasnul Hadi, Pin Jern Ker, Hui Jing Lee, Yang Sing Leong, Mahammad A. Hannan, Md. Zaini Jamaludin, Mohd Adzir Mahdi

**Affiliations:** 1 Institute of Sustainable Energy, Universiti Tenaga Nasional, Kajang 43000, Malaysia; se23032@utn.edu.my (M.H.H.H.); hannan@uniten.edu.my (M.A.H.); 2 Institute of Power Engineering, Universiti Tenaga Nasional, Kajang 43000, Malaysia; LHjing@uniten.edu.my (H.J.L.); p107849@siswa.ukm.edu.my (Y.S.L.); mdzaini@uniten.edu.my (M.Z.J.); 3 Department of Electrical, Electronic and System Engineering, Faculty of Engineering and Built Environmental, Universiti Kebangsaan Malaysia (UKM), Bangi 43600, Malaysia; 4 Wireless and Photonics Networks Research Centre, Faculty of Engineering, Universiti Putra Malaysia (UPM), Serdang 43400, Malaysia; mam@upm.edu.my

**Keywords:** ASTM D1500, color, insulating oil, power transformers, single wavelength, transformer oil, ultraviolet-blue wavelength

## Abstract

The color of transformer oil can be one of the first indicators determining the quality of the transformer oil and the condition of the power transformer. The current method of determining the color index (CI) of transformer oil utilizes a color comparator based on the American Society for Testing and Materials (ASTM) D1500 standard, which requires a human observer, leading to human error and a limited number of samples tested per day. This paper reports on the utilization of ultra violet-blue laser at 405- and 450-nm wavelengths to measure the CI of transformer oil. In total, 20 transformer oil samples with CI ranging from 0.5 to 7.5 were measured at optical pathlengths of 10 and 1 mm. A linear regression model was developed to determine the color index of the transformer oil. The equation was validated and verified by measuring the output power of a new batch of transformer oil samples. Data obtained from the measurements were able to quantify the CI accurately with root-mean-square errors (RMSEs) of 0.2229 for 405 nm and 0.4129 for 450 nm. This approach shows the commercialization potential of a low-cost portable device that can be used on-site for the monitoring of power transformers.

## 1. Introduction

Power transformers are key assets of power utilities that ensure the regulation and distribution of electricity to housing and industrial areas. The insulation system is constantly exposed to electrical stress, mechanical stress, and thermal stress [1,2,3,4] during its operation. Lack of supervision of the condition of the transformer may cause catastrophic failures. In this regard, regular maintenance and monitoring of its insulation system are important to ensure their functionality is in an optimum condition. Conventionally, transformer oil is sampled from the power transformers and sent to an accredited laboratory for test analysis.

Early detection of transformer oil degradation is important through its color visualization since the quality of the transformer oil can be reflected by its color [5]. Color changes may be indicative of problems in the production process, contamination, degradation, or the oxidation of the materials and products. In addition, as the level of degradation of the transformer oil increases, the color of the transformer oil becomes darker [4]. Therefore, a faster approach for color measurement, which is cost effective and has high accuracy, is required.

There are several available methods for measuring the color of liquid, such as visual examinations [6], color comparator [7], visual colorimeter [8,9,10], automatic colorimeter [11,12], optical spectroscopy [13,14,15,16,17,18,19,20,21,22,23,24,25], and image analysis [26,27,28,29,30,31,32]. The conventional method of measuring the color of transformer oil is to use a color comparator, where a sample is compared with a standard colored disc. The American Society for Testing and Materials (ASTM) D1500 is a standard color scale and test method for ASTM color of petroleum products including transformer oil. The ASTM color scale consists of 16 ASTM color indices ranging from 0.5 for the lightest color to 8.0 for the darkest color, with a 0.5 step size [7]. Operation wise, using a light source with a color temperature of 2750 K, the sample in a standard glass jar is placed in the comparator, and it is compared with colored glass discs of the 16 ASTM color. If the color of the sample matches with any of the color disc, the color is reported. Otherwise, if the sample color is between two ASTM colors, the darker glass of the ASTM color preceded by the letter “L” is reported.

The optical spectroscopy technique has received increasing interest from researchers and industries as it is a non-destructive method. Through optical techniques, it eliminates human handling error and improves the measurement accuracy [13]. Researchers have used this method for measuring the color of palm oil [33,34], olive oil [25,35,36,37,38], honey [18,19,20,39], maple syrup [40,41], beer [21,22,42], and vegetable oils [15,16,43]. Specifically for transformer oil, Leong et al. [13] demonstrated the possibility of determining the color index (CI) of transformer oil in accordance with ASTM D1500 using ultraviolet (UV)-visible spectroscopy by measuring its optical absorbance at wavelengths from 300 to 700 nm. The study shows that different color index of transformer oil can be accurately identified in the UV-visible waveband.

Although the UV-visible waveband has been used widely in the industries to measure the color of various types of oils, the basis of utilizing a single wavelength or a combination of a few wavelengths for color measurement of olive oil was first studied and reported by D. Escolar et al. [37]. Two absorbance measurements at 480 and 670 nm from a spectrophotometer were used to develop a mathematical model. The mathematical model was then used to estimate the chromatic coordinates and the chroma of olive oil based on the International Commission on Illumination (CIE) Lab values. Although this method does not exactly utilize a single-wavelength light source, the concept of measuring color using a single wavelength was exercised. Subsequently, R. Sanga et al. [44] managed to develop an in-line quasi-digital sensor system. This system utilizes two single-wavelength light-emitting diodes (LEDs) at 590 and 840 nm to measure the color and turbidity of lubricant oil. Two sets of optical systems were designed using two sets of light-dependent resistors (LDRs) and LEDs. One set was used to measure the color of the lubricant oil while another set was used to measure the turbidity and to correct the deviation in the color scale reading due to turbidity. An embedded system is required to convert the pulse frequency into the color scale. In comparison to other works on the utilization of a single wavelength for the determination of color, this system uses an LED, which has a broader spectrum across a wider waveband compared to a laser diode.

**Figure 1:**
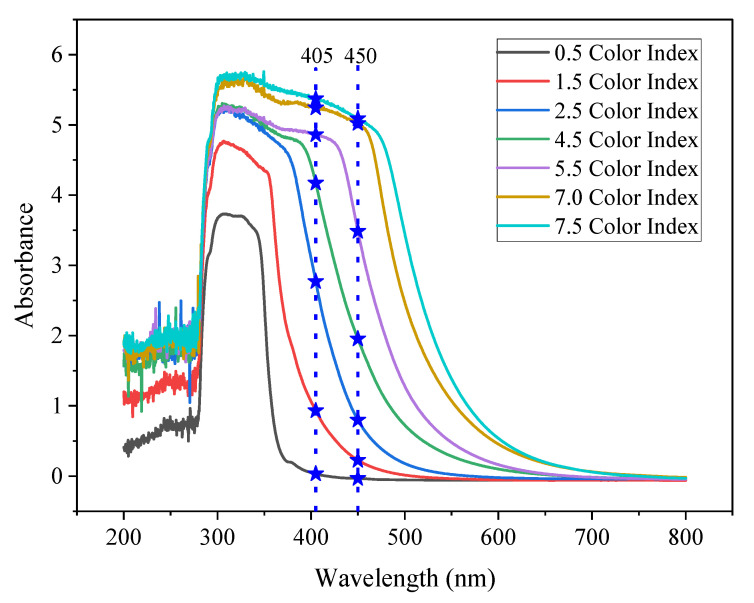
Optical absorbance spectrums for transformer oil samples based on ASTM D1500 and the possibility of using a single-wavelength laser diode.

Previous works have shown that color measurement using a single wavelength is achievable. However, there is still no study on the color measurement of transformer oil using a single-wavelength laser diode in the UV-blue wavelength range. Figure 1 shows the absorbance spectrum for transformer oil samples with different color indices obtained from the measurement conducted by Leong et al. [13]. The measurement was carried out by using an ultraviolet-visible-near infrared (UV-Vis-NIR) spectrophotometer. They developed a mathematical model to determine the color index of transformer insulating oil using UV-visible spectroscopy with reference to the ASTM D1500 standard. Each color index has a different absorbance value throughout the UV-visible wavelength range. The color index is directly proportional to the absorbance value.

It was observed that in the UV-blue waveband (400–500 nm), the absorbance values of the oil samples with different color indices are distinctly different. The blue dashed line shows the possibility of using a single-wavelength laser diode to determine the color index of transformer oil. The points on the blue dashed line that intercepts on the absorbance graph shows that each color index can be differentiated and measured using a single wavelength. Considering the availability of laser diodes at certain wavelengths, laser diodes at wavelengths of 405 and 450 nm were chosen as the light source because the absorbance of the oil sample can be distinctively determined.

Therefore, a comprehensive study was carried out to investigate the absorption of transformer oil samples with various color indices at wavelengths of 405 and 450 nm. The correlation between the color index in accordance with the ASTM D1500 standard and the optical power output was established, and mathematical models were formulated. Critical comparisons between the proposed method and the other techniques were provided. The contributions of the study are as follows:

1. Utilization of a single-wavelength laser diode in the UV-blue wavelength for color index measurement of transformer oil was established.
2. Mathematical models were developed and validated to correlate the output power with the color index in accordance with ASTM D1500.

## 2. Experimental Details

**Figure 2:**
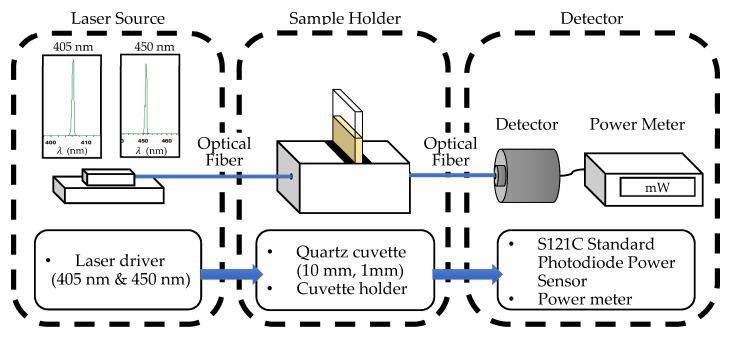
Schematics diagram of the components of the optical measuring setup.

In this study, transformer oil samples were collected, and their color indices were measured using a color comparator in accordance with the ASTM D1500 by an accredited lab. The oil samples were then tested, where the light beam from the single-wavelength laser diode passed through the oil sample and was detected by a photodiode sensor. The experimental setup for this study is shown in Figure 2. Light from the laser source was transmitted through the oil sample, which was placed in a quartz cuvette. The resulting optical signal was detected by the photodiode sensor. Table 1 shows the details and parameters of the components used in this experiment.

As shown in Figure 2, the setup is divided into three parts. The first part is the laser source. The laser diode is connected by fiber optic cable to the cuvette holder. Two commercially available laser diodes were used for the measurements and data collection. To ensure the consistency of the laser power, the laser diode was initially warmed up for 30 min at a constant operating temperature of 25 °C before conducting the measurement. Both laser diodes were operated at the same operating voltage of 3.3 V. For the 405-nm laser diode, the output power was 20 mW while the output power of the 450-nm laser diode was 50 mW. This difference in the output powers of the two laser diodes is due to the availability of the laser diode in the market. Although the output powers were different, measurements were made to observe whether higher output power can affect the results of the color measurement of transformer oil.

The second part of the setup is the sample holder. The sample holder is where the oil sample is located and is placed in a cuvette holder. Quartz cuvette cells with two different optical pathlengths of 10 and 1 mm were used in this experiment. This is to compare and to minimize the effect of optical pathlengths on the accuracy of the color index measurement. Before conducting the experiment, the oil sample was slowly pipetted into the cuvette cell to prevent the formation of bubbles. To ensure the consistency of the measurement, the sides of the cuvette were cleaned, such that there was no dust or fingerprints on the cuvette to allow optimum light interaction with the transformer oil samples.

In this experiment, 20 transformer oil samples with a color index ranging from 0.5 to 7.5 were used. However, a limited number of samples of only 1 or 2 oil samples were obtained for color index 4.5 and above. This is because the oil collected was from operating power transformers, where, according to the IEC 60422 standard, the oil needs to be replaced or maintained after reaching a certain threshold of color index. For the output power detection, a silicon photodiode was used. The amount of light detected by the sensor was measured using an optical power meter. To ensure the consistency of the reading collected, 5 readings were recorded at 10-s intervals. The average of the 5 readings was computed with a maximum error from ±0.07% to ±1.50%.

## 3. Results and Discussion

**Figure 3:**
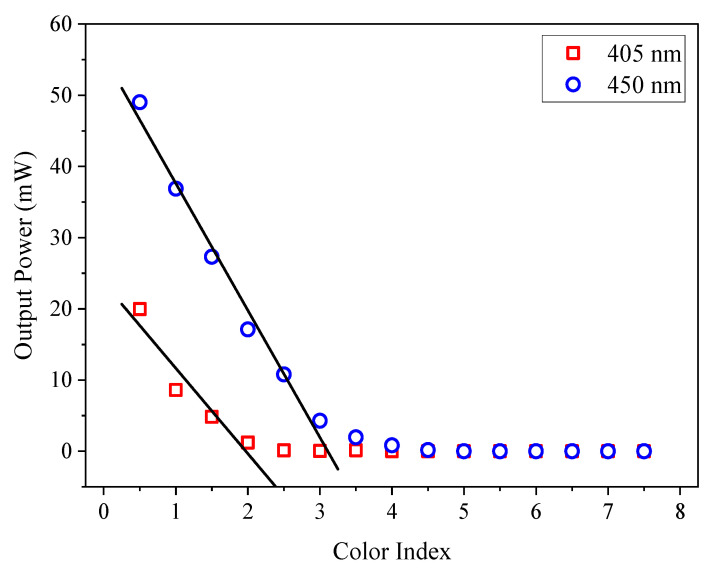
Measurement using laser diodes at wavelengths of 405 (input power = 24.7 mW) and 450 nm (input power = 55.8 mW) with a 10-mm pathlength cuvette.

Figure 3 shows the output power at wavelengths of 405 and 450 nm when oil samples with different color indices were measured using the 10-mm pathlength cuvette. Based on the collected data, the output power decreases as the color index of the oil sample increases. The reduction of the output power, either at 405 or 450 nm, was due to the optical absorption by the oil sample. The steeper decreasing slope for the measurement at 405 nm indicates that the measurement of the color index at this wavelength is more sensitive, compared to the measurement at 450 nm. For the measurement at 405 nm (450 nm), the output power saturated to almost zero for CI ≥ 2 (CI ≥ 3.5).

This saturation corresponds to the Beer–Lambert’s law [45], where the loss of light intensity is directly proportional to the absorbance and length of the light path. In this case, the length of the light path, which is the cuvette pathlength, is long enough for most of the light to be absorbed by the transformer oil samples. Thus, this causes the output power to be saturated. The saturated CI was discarded because it does not represent the real measured values and only measurements up to CI = 1.5 for 405 nm, and CI = 3.0 for 450 nm were used for the linear regression. A linear decrement relationship can be observed between the color index and the output power when the saturated data was excluded. To measure the strength of the correlation between the variables, the Pearson product-moment correlation coefficient (*r*) of the data was calculated. The *r* value indicates the strength and the trend line direction of the linear relationship between the two variables. The calculated *r* values between the color index and output power in Figure 3 is −0.9539 for 405 nm and −0.9925 for 450 nm. Table 2 shows the guideline to determine the strength of the correlation relationship for absolute value of *r* (|*r*|). In both cases, since |*r*| > 0.95, the strength of linear correlation is considered very strong.

The negative *r* value indicates a negative correlation between the color index and output power. The two correlation coefficient show a very strong linear correlation as the absolute values of *r* are very close to 1.

Although measurement using 10 mm was saturated at a certain CI, a linear regression line was done on the unsaturated data points and the regression model was used to calculate the CI. Table 3 shows the linear equations obtained from the regression model.

Nevertheless, to ensure that the measurement can be done for the full range of CI (0.5 to 7.5) based on the ASTM D1500 standard, a cuvette with a shorter pathlength of 1 mm was used. The interaction of light with the oil sample was shortened, thus reducing the light absorbance.

**Figure 4:**
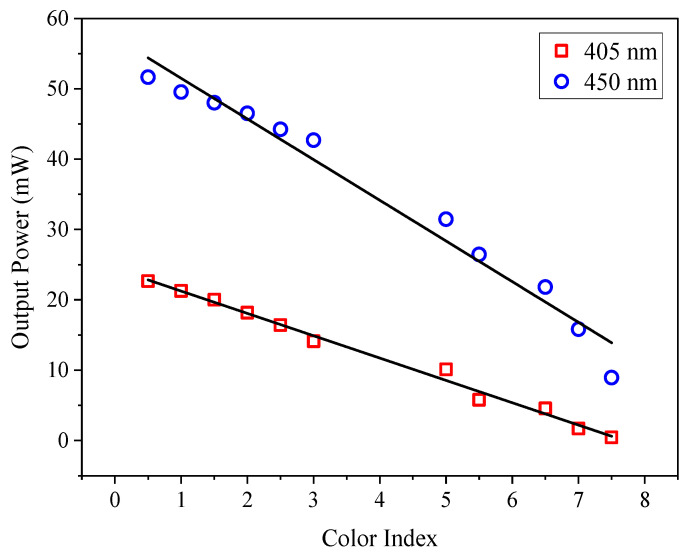
Measurement using the 405-nm laser diode (input power = 24.4 mW) and 450-nm laser diode (input power = 54.7 mW) with a 1-mm pathlength cuvette.

Data collected from the measurement using the 1-mm pathlength cuvette are plotted in Figure 4. The *r* values for the data points in Figure 4 were also calculated. The *r* values for the data points of 405 and 450 nm are −0.99958 and −0.986, respectively, which also show a very strong linear correlation. The data points for the measurements were fitted with a linear regression model that described the relationship of the data. The linear regression model obtained from the plotted data points for the CI measurement at 405 and 450 nm is shown in Table 4.

The equations were rearranged as follows to determine the color index:

(1) $$CI_{405}=\frac{Output\;Power-24.421}{\left(-3.1745\right)}$$

(2) $$CI_{450}=\frac{Output\;Power-57.285}{\left(-5.7832\right)}$$

where *x* is the *CI* and *y* is the *Output Power* measured.

The linear regression line on the data obtained from measurements at 405 nm has an R^2^ value of 0.99168, while measurement with the 450-nm laser diode has an R^2^ value of 0.97202. Based on Table 4, the slope for 450 nm is steeper than 405 nm. The steeper slope indicates that the absorbance value increased more significantly at 450 nm as the color index increased. However, the data distribution measured at 405 nm has better linearity compared to that measured at 450 nm. This was because the absorbance value increased consistently with the increasing color index as shown in Figure 1. At 450 nm, the increment in the absorbance value with the increasing color index was not consistent. The increments in the absorbance value for low (0.5 to 1.5) and high (7.0 and 7.5) color indices were smaller, compared to those at 2.0 ≤ *CI* ≤ 6.5. Therefore, 405 nm gave a better regression line fitting, which led to a better R^2^ value.

To validate the mathematical models of Equations (1) and (2), a new set of transformer oil samples (S1–S11) were collected from the accredited laboratory, with their results on the color indices in accordance with the ASTM D1500 standard. However, since a transformer oil sample with a higher color index was difficult to obtain, a few samples were reused to validate the equation. The repeating samples were S8–S11. The oil samples were measured for their output power at 405 and 450 nm with a 1-mm pathlength cuvette. The estimated *CI* was calculated using Equations (1) and (2). The difference between the actual *CI* obtained from the ASTM D1500 measurement and the estimated *CI* using the developed model was then calculated and analyzed.

Based on Table 5, the result shows that the estimated *CI* for each sample using Equation (1) was closer to the actual *CI* compared to the estimated *CI* using Equation (2). The root-mean-square error (RMSE) values were calculated and compared for Equations (1) and (2). RMSE defines the standard deviation of the difference between the actual value and estimated value. The error from RMSE was possibly due to the measurement process, and the model developed. The error could also be due to the measurement from the conventional method of the ASTM D1500 standard. Through RMSE, the variation of the actual data near the regression line can be identified.

Theoretically, based on Figure 1, measurement at 450 nm should be able to predict the *CI* for samples with high *CI* more accurately than measurement at 405 nm. This is because the differences in absorbance for the higher color index at 450 nm is bigger than that at 405 nm. However, the difference in *CI* for S5, S6, S7, S9, S10, and S11 in Table 5 showed otherwise as the estimated *CI* using Equation (2) has a bigger difference in *CI*. It is also shown that the RMSE for Equation (1) is 0.2229, which is lower compared to RMSE for Equation (2), which has an RMSE of 0.4129. Compared to the ASTM D1500 standard, which has an error of ≤0.5 due to the method of reporting, the RMSE of 0.4129 using Equation (2) is within the acceptable range of error, although it was higher than that obtained from Equation (1).

The RMSE value (0.2229) obtained from this work was slightly higher compared to the RMSE (0.1961) obtained by Leong et al. [13]. However, this work utilized data obtained from a single wavelength while Leong et al. [13] relied on data obtained from 350 to 700 nm and correlated the *CI* with the cutoff wavelengths from the absorbance spectrum and absorbance of the oil samples. The conventional method of measuring the color index according to ASTM D1500 relies on manual visual inspection by an operator, which limits the number of measurements per day. This conventional method also depends on the operator’s perception of colors, which can lead to human error. Other than that, the color scale contains a large step size of 0.5 *CI*, which can result in higher error of the color index. This shows that measurement using a single-wavelength laser diode produced a sufficiently small error with a simpler measurement setup.

To elucidate the effect of the optical pathlength on the accuracy of *CI* measurement, repeating samples were used for the validation of the regression models (refer to Table 3 and Table 4) using 10- and 1-mm cuvettes. The estimated *CI* values using the regression models are shown in Table 6. The measurement at both wavelengths using the 10-mm pathlength showed good RMSE values of 0.1181 and 0.1055.

A comparison between measurements using the 10- and 1-mm pathlengths is shown in Table 6. Repeating samples were used for this comparison. At 450 nm, the utilization of the 10-mm pathlength cuvette improved the RMSE significantly from 0.3309 to 0.1055. However, the RMSE obtained for measurements at 405 nm with the 10- and 1-mm pathlengths were comparable. This shows that measuring different *CI* (from 0.5 to 8.0) at the optimum optical pathlength is important in minimizing the measurement error.

Table 7 presents a summary of the methods and techniques for color index measurement of transformer oil. The results demonstrated that the utilization of a single-wavelength laser diode in the UV-blue wavelength in determining the *CI* of transformer oil based on ASTM D1500 can provide better sensitivity up to 0.1 CI. Human handling error can also be eliminated as it does not require a human observer for the color identification process. In comparison with previous work that utilizes the full spectrum from 350 to 700 nm, this work requires only a single-wavelength optical source, which simplifies the optoelectronic components significantly.

## 4. Conclusions

This work shows that the color index of transformer oil can be measured using a single-wavelength optical source. Based on the ASTM D1500 standard, linear regression models were developed to accurately determine the color index of transformer oil using the data obtained from measurements at 405 and 450 nm. Model validation using a second batch of oil samples showed that the models were able to determine the color index accurately, with RMSE values of 0.2229 and 0.4129 for data measured at 405 and 450 nm, respectively. The results of this work demonstrate that UV-blue wavelengths at 405 and 450 nm can be used for the determination of the color index of transformer oil. Unlike previous work that requires a spectrophotometer for measurements of the full spectrum from 350 to 700 nm, the utilization of a single wavelength promises a much-simplified portable device for the color measurement of transformer oil. Although the measurement using either 405 or 450 nm can provide accurate color measurement of transformer oil, it is hypothesized that a hybrid of both wavelengths may lead to better accuracy. While this work has clearly demonstrated the possibility of utilizing single-wavelength measurement to determine the color index of transformer oil, the following future works are proposed to achieve higher accuracy:

1. A detailed study on the effect of optical pathlength variation and more accurate color index measurement.
2. An investigation of the optimum laser power required for a particular color index to ensure that transformer oil with the full range of the color index can be measured.
3. Due to the variations of optical pathlengths and optimum laser powers, a machine learning-based model can be developed to more accurately model the color index of transformer oil based on multiple inputs.

## Figures and Tables

**Table 1:** Components’ details and parameters.

Component	Details	Parameters
Laser Diode	405 nm	Output Power: 20 mW
	450 nm	Output Power: 50 mW
Quartz Cuvette	10 mm	Volume: 3.5 mL
	1 mm	Volume: 0.35 mL
Detector	Thorlabs S121C Standard Photodiode Power Sensor	Material: Silicon Range of Detection: 400 nm to 1100 nm Responsivity: <1 μs Sensitivity: 10 nW

**Table 2:** The interpretation of the strength of the relationship for absolute values of correlation.

Absolute Value of *r*, |*r*|	Strength of Relationship
0–0.19	Very weak
0.20–0.39	Weak
0.40–0.59	Moderate
0.60–0.79	Strong
0.80–1.00	Very strong

**Table 3:** Linear equation and R^2^ for measurement using the 10-mm pathlength cuvette.

Wavelength (nm)	Equation	Intercept, *a*	Slope, *b*	R^2^
405	*y = a + b × x*	26.269	−15.133	0.92277
450		55.438	−17.830	0.98497

**Table 4:** Linear equation and R^2^ for measurements using the 1-mm pathlength cuvette.

Wavelength (nm)	Equation	Intercept, *a*	Slope, *b*	R^2^
405	*y = a + b × x*	24.421	−3.1745	0.99168
450		57.284	−5.7831	0.97202

**Table 5:** Comparison between ASTM D1500 standard, and estimated color index using Equations (1) and (2).

	Sample	ASTM D1500	Estimated CI	Difference in CI	RMSE
Equation (1) *CI*_405_ (R^2^ = 0.99170)	S1	0.5	0.55	0.05	0.2229
	S2	1.0	0.99	−0.01	
	S3	1.5	1.39	−0.11	
	S4	2.0	1.97	−0.03	
	S5	2.5	2.52	0.02	
	S6	3.0	3.25	0.25	
	S7	5.0	4.50	−0.50	
	S8	5.5	5.87	0.37	
	S9	6.5	6.26	−0.24	
	S10	7.0	7.15	0.15	
	S11	7.5	7.55	0.05	
Equation (2) *CI*_450_ (R^2^ = 0.97200)	S1	0.5	0.97	0.47	0.4129
	S2	1.0	1.34	0.34	
	S3	1.5	1.60	0.10	
	S4	2.0	1.86	−0.14	
	S5	2.5	2.25	−0.25	
	S6	3.0	2.52	−0.48	
	S7	5.0	4.46	−0.54	
	S8	5.5	5.33	−0.17	
	S9	6.5	6.13	−0.37	
	S10	7.0	7.17	0.17	
	S11	7.5	8.36	0.86	

*CI*—Color index.

**Table 6:** Comparison of the estimated *CI* between the 10-mm pathlength cuvette and 1-mm pathlength cuvette.

Wavelength	Sample	ASTM D1500	Cuvette					
			10 mm			1 mm		
			Estimated *CI*	Difference	RMSE	Estimated *CI*	Difference	RMSE
405	S1	0.5	0.42	−0.08	0.1181	0.55	0.05	0.0728
	S2	1.0	1.17	0.17		0.99	−0.01	
	S3	1.5	1.42	−0.08		1.39	−0.11	
450	S1	0.5	0.36	−0.14	0.1055	0.97	0.47	0.3309
	S2	1.0	1.04	0.04		1.34	0.34	
	S3	1.5	1.58	0.08		1.60	0.10	
	S4	2.0	2.15	0.15		1.86	−0.14	
	S5	2.5	2.50	0.00		2.25	−0.25	
	S6	3.0	2.87	−0.13		2.52	−0.48	

**Table 7:** Comparison of methods for color index measurement of transformer oil.

Methods	Wavelength (nm)	Human Observation	Model Equation	Accuracy
ASTM D1500 standard	NA	✓	✕	Max. error of 0.5 is tolerated
UV-Vis Spectroscopy	300–700	✕	✓	RMSE = 0.1961
Single wavelength spectroscopy	405	✕	✓	RMSE_405_ = 0.2229
	450	✕	✓	RMSE_450_ = 0.4129

## Data Availability

Not applicable.
